# Fracture Resistance of Ceramic Laminate Veneers Bonded to Teeth with Class V Composite Fillings after Cyclic Loading

**DOI:** 10.1155/2018/1456745

**Published:** 2018-04-17

**Authors:** Leyla Sadighpour, Farideh Geramipanah, Vanya Rasaei, Mohammad J. Kharazi Fard

**Affiliations:** ^1^Department of Prosthodontics, Faculty of Dentistry, Tehran University of Medical Sciences, Tehran, Iran; ^2^Dental Implant Research Center, Faculty of Dentistry, Tehran University of Medical Sciences, Tehran, Iran; ^3^Dental Research Center, Faculty of Dentistry, Tehran University of Medical Sciences, Tehran, Iran

## Abstract

**Purpose:**

Porcelain laminate veneers (PLVs) are sometimes required to be used for teeth with composite fillings. This study examined the fracture strength of PLVs bonded to the teeth restored with different sizes of class V composite fillings.

**Materials and Methods:**

Thirty-six maxillary central incisors were divided into three groups (*n*=12): intact teeth (control) and teeth with class V composite fillings of one-third or two-thirds of the crown height (small or large group, resp.). PLVs were made by using IPS e.max and bonded with a resin cement (RelyX Unicem). Fracture resistance (*N*) was measured after cyclic loading (1 × 10^6^ cycles, 1.2 Hz). For statistical analyses, one-way ANOVA and Tukey test were used (*α*=0.05).

**Results:**

There was a significant difference between the mean failure loads of the test groups (*P*=0.004), with the Tukey-HSD test showing lower failure loads in the large-composite group compared to the control (*P*=0.02) or small group (*P*=0.05). The control and small-composite groups achieved comparable results (*P* > 0.05).

**Conclusions:**

Failure loads of PLVs bonded to intact teeth and to teeth with small class V composite fillings were not significantly different. However, extensive composite fillings could compromise the bonding of PLVs.

## 1. Introduction

Porcelain laminate veneers (PLVs) represent an excellent treatment option for restoring aesthetics while preserving the integrity of the remaining tooth structure. Its clinical acceptance is mainly due to minimal preparation, successful clinical outcomes, and high patient satisfaction [1]. The estimated survival rate of PLVs has been reported to be 94.4% after five years and 93.8% after 10 years [2]. The most frequent causes of failure, including microleakage, debonding, and fracture, are related to the quality of the bonding surface and the choice of the luting agent [3, 4]. Because the retention of PLVs relies solely on the bond to the tooth structure, the availability of sufficient enamel has been considered as a critical factor for its long-term success [5]. For a predictable result, it is recommended that at least 50% of the labial enamel or intraenamel margins be present [6, 7]. The amount of enamel may be compromised by several factors, such as fracture, wear, and preparation of various types of restorations, including composite fillings [8–10]. Even standard preparations for PLVs could result in serious enamel damage. Various preparation techniques could compromise 30% to 50% of the labial surface enamel, leading to dentin exposure [11, 12].

Patients commonly seek esthetic treatment for discoloured or aged composite fillings [7, 13]. One study on the clinical performance of PLVs showed that 70% of patients had previous composite fillings [2]. Under such circumstances, some clinicians believe that the preparation margins should be extended to sound enamel, far to the interproximal or even to the lingual enamel, to create intraenamel margins [7, 8]. Findings from several clinical studies support this approach. For example, higher failure rates were observed when laminate veneers were bonded to large-composite fillings [2, 3]. A reliable bonding could be achieved with greater quantities of enamel surface and margins. However, this process compromises excess dental hard tissue [13, 14].

An alternative approach is to bond PLV onto an acceptable composite filling [15, 16]. Gurel et al. [12] found no relation between the existing restorations and PLV failure. Similarly, Gresnigt et al. [17] found no significant difference in the 40-month clinical performances of PLVs bonded to teeth with and without existing composite fillings. Moreover, the overall survival rate remained high, at 94.6%. Although the available literature suggests that the size, age, and location of composite fillings are among the factors that may influence the failure rate of PLVs bonded to existing restorations, these factors have not been clearly identified over literature. Quantifying the amount of enamel that can be replaced by composite fillings without affecting the fracture resistance may improve our clinical decision-making. Few investigations have been conducted with a focus on the performance of PLVs when bonded to composite fillings with defined dimensions [16–18]. In an in vitro study, Sadighpour et al. [18] found that fracture resistance of PLVs bonded to intact teeth or teeth with a classic class III composite filling was similar. In addition, fracture resistance decreased and microleakage of PLVs increased when the composite fillings were removed and replaced by veneers. Class V composite fillings could differ from class III fillings in location, size, and stress patterns [19]. Furthermore, a typical class V cavity could compromise a greater amount of labial enamel than either a class III or IV cavity that has not been investigated.

In the light of this background, the aim of the present study was to examine the fracture strength of PLVs bonded to teeth restored with different sizes of class V composite fillings. The null hypothesis was that the fracture strength of teeth restored with PLVs would not be affected by the presence of class V composite fillings of different sizes.

## 2. Materials and Method

The sample for this study included 36 human maxillary central incisors that had been extracted from adults within the past three months due to periodontal problems. All patients provided informed consent for extraction and subsequent use of their teeth for experimental study, according to the protocol of the Clinical Research Ethics Board at Tehran University of Medical Sciences. Only teeth with no caries, cracks, or excessive wear were included in the study.

Teeth were cleaned of tissue tags and debris by using a hand instrument and were stored in 1% chloramine for two weeks and then in distilled water until use. The teeth fell into three groups based on the labial surface area (65 ± 5 mm, 75 ± 5 mm, and 85 ± 5 mm).

Teeth from each size group were randomly assigned to three groups: control (NC) (intact with no composite fillings), small-composite (SC), and large-composite (LC) (*n*=12).

In the NC group, the incisal edges were reduced by 1 mm. Three orientation grooves were made on the labial surface by a 0.3 mm cutting-depth diamond bur (868B.314.018, Komet Dental, Lemgo, Germany) and were joined together by a round-end diamond bur (868.018, Komet Dental). Cervical finish lines were ended 1 mm above the cementoenamel junction (CEJ) to ensure intraenamel margins. Proximally, the reduction was extended by 1 mm toward the lingual surface. All line angles were rounded, and the margins were finished.

**Figure 1:**
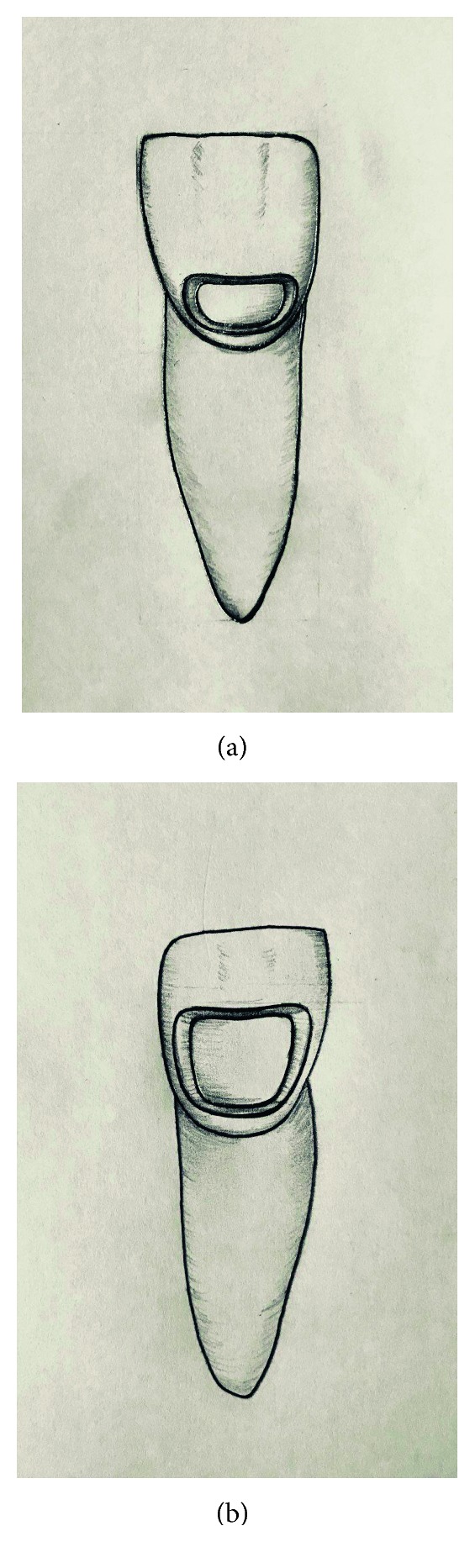
(a) Representative of a small class V cavity (b) and large class V cavity.

**Figure 2:**
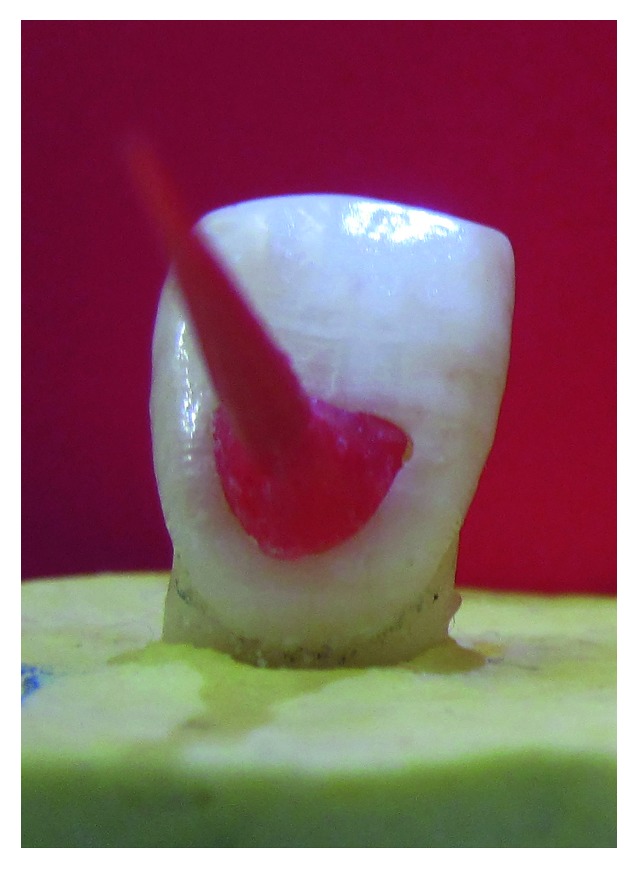
Resin template for guiding the cavity preparation.

**Figure 3:**
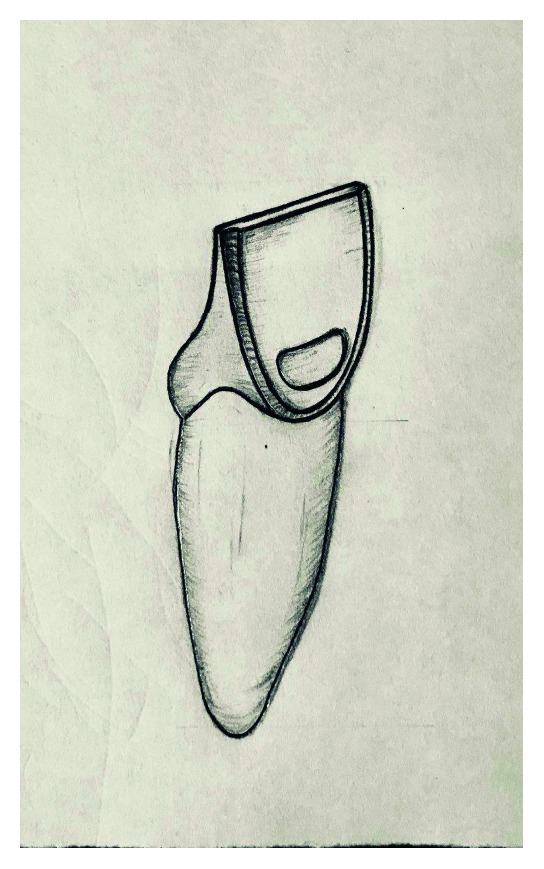
Schematic representation of final preparation.

In SC and LC groups, a class V cavity at one-third or two-thirds of the crown height, respectively, was prepared for composite filling. Each cavity was 1 mm short of the proximal finish line and 2 mm short of the CEJ, Figure 1. Autopolymerised acrylic resin (GC Pattern Resin, GC America Inc., Alsip, IL, USA) was used in one cavity each from the SC and LC groups to fabricate a template for the remaining teeth, Figure 2. Cavities were etched for 15 s with 38% phosphoric acid (Etching Gel, Faghihi Dental Co., Tehran, Iran), rinsed for 15 s, and then gently air dried. An etch-and-rinse dentin bonding agent (Single Bond, 3M ESPE, MN, USA) was applied for 30s and light polymerised (Bluephase 16i, Ivoclar/Vivadent, Schaan, Liechtenstein) with a 5 s burst at 1100 mW/cm^2^. Cavities were restored by using a universal microhybrid composite resin (Filtek Z250, 3M ESPE), which was inserted in two layers and light polymerised with an LED light unit (Bluphase 16i). Teeth were prepared for PLV in a similar way as in the NC group, Figure 3. Specimens were stored in water at room temperature of 25°C for three weeks before receiving veneers. A custom tray was fabricated with autopolymerising acrylic resin (Tray Material, Major, Moncalieri, Italy) for each specimen. Impressions were made by a polyvinyl siloxane material (Monopren, Kettenbach GmbH, Eschenburg, Germany) and poured with type IV dental stones (Fuji Rock, GC Corp., Tokyo, Japan). PLVs were fabricated with a lithium disilicate ceramic (IPS e.max Press, Ivoclar/Vivadent) using the lost-wax technique. Wax patterns were fabricated at a uniform thickness of 0.7 mm, sprued with four patterns per cylinder, invested with a phosphate-bound investment (IPS Pres/Vest, Vivadent/Ivoclar), and burned out in a conventional porcelain-firing furnace (Vaccumat 300, Vita, Zahnfabrik H. Rauter GmbH, Bad Sackingen, Germany) at 850°C. Low-translucency ceramic ingots in shade A2 were pressed in a Programat furnace (Ivoclar/Vivadent) at 1050°C. Molds were divested by using 50 *µ*m aluminium oxide air particles at 0.4 MPa and a distance of 10 mm for 5 s and then cleaned in an ultrasonic cleaner (Prosonic 300, Sultan Health Care, Englewood, NJ, USA).

PLVs on teeth were investigated for any cracks or flaws under 5x magnification, and the fit was examined on the corresponding dies. PLVs were glazed (IPS e.max Ceram Glaze, Vivadent/Ivoclar) in a conventional porcelain-firing furnace (Vacumat 300) at 750°C. PLVs were etched with 9.5% hydrofluoric acid (Porcelain Etchant, Bisco Inc., Schaumburg, IL, USA) for 90s, cleaned under water for 30 s and then silanized (Monobond S, Vivadent/Ivoclar). Enamel surfaces and margins were etched by 37% phosphoric acid (Etching Gel) for 15 s and washed for 15 s. A self-adhesive resin cement (RelyX Unicem, Automix, 3M/ESPE, St Paul, MN, USA) was applied on the bonding surface of veneers and then seated on the tooth surface and held in place with finger pressure for 10 s. The excess cement was removed by a scalpel blade. Light polymerisation (Bluephase 16i, Vivadent/Ivoclar) was performed for 10 s. The specimens were stored for 24 h in water at room temperature before being subjected to thermal cycling (2000 thermal cycles between 5°C and 55°C, with a dwell time of 30 s in each bath and a transfer time of 10 s). The periodontal ligament was simulated with polyether elastomer (Impergum F, 3M/ESPE) on the roots of the teeth before being mounted on a chewing simulator. Specimens were mounted at 135° with the contact point on the cingulum. To simulate three years of service, specimens were subjected to 1.2 × 10^6^ cycles of 50 N loads at a frequency of 2 Hz.

Fracture resistance was measured by applying a static load to the specimens until fracture using a universal testing machine (Zwick Roell Z050, Ulm, Germany). A compressive load was applied at 1 mm/min to the incisal edge of the specimens with a stainless-steel sphere, 8 mm in diameter, and a thin sheet in between the contact points. The fracture load was automatically recorded as the fracture resistance of each PLV.

The failure mode was examined with a stereomicroscope (Zeiss OPM1, Carl Zeiss, Oberkochen, Germany) at a magnification of ×20. The failure mode was categorised as adhesive (failure between the tooth and laminate), cohesive (failure within the laminate or tooth), or mixed. The homogeneity of variances and normal distribution of data were verified by Levene's test and the Kolmogorov–Smirnov test, respectively. Then, the mean values of fracture resistance were statistically analysed by one-way ANOVA and Tukey-HSD post hoc using SPSS (SPSS Inc., Chicago, IL, USA) at a significance level of 95% (*α*=0.05).

## 3. Results

During cyclic loading, one specimen each in the NC and SC groups and two specimens in the LC group were lost due to fracture. Fracture value for the lost specimens was set to zero.

The descriptive data of fracture resistance are summarised in Table 1. The mean failure loads were significantly different among the test groups (*P*=0.004 by one-way ANOVA). Tukey-HSD revealed that the failure load in the LC group was lower than the failure load in the NC or SC group (*P*=0.02  and  0.05, resp., by Tukey's test). Adhesive failure mode was dominant in the LC group, and cohesive failure occurred more often in the NC and SC groups (Table 2).

## 4. Discussion

The mean fracture resistance of the PLV-restored teeth with class V composite fillings was examined after cyclic loading. No difference in fracture resistance was found between teeth with composite filling to one-third of the crown height (small-composite group) compared to teeth with an intact labial surface (control). However, the mean fracture resistance was significantly lower in the large-composite group compared to the small-composite or control group. Therefore, the null hypothesis should be partially accepted.

In this study, teeth with 33% of the original enamel remaining (one-third) on the labial surface showed the lowest fracture strength among the groups. Gurel et al. [12] have also emphasised on the role of the remaining enamel on the failure rates of PLVs. They found that PLVs bonded to the dentin had 10 times greater risk of failure than PLVs bonded to the enamel. In contrast, the presence of composite fillings was not correlated with an increased risk of failure, and PLVs bonded to composite fillings had outcomes that were more favourable. However, excessive dentinal exposure, dentinal margins, and the size of composite fillings were not quantified by the authors. Therefore, a direct comparison could not be performed with our study. Contrary to the findings of our study, Gresnigt et al. [17] found that the size of composite fillings did not affect the survival rate of PLVs over a period of 40 months. The size categories in their study were close to those in the small- and large-composite groups of our study. The chairside application of surface silica coating on the resin composite surface before PLV bonding may explain the discrepancy between the studies. Furthermore, the overall follow-up time of their study was short, and most failures occur after years of service [3, 7]. In the present study, fatigue ageing was performed by applying cyclic loading in an attempt to simulate a three-year clinical service.

The luting agent is another contributing factor to the longevity of adhesive restorations [4]. We used a self-adhesive cement, which allows bonding to a range of substrates without pretreatment. The multiple steps of etching, rinsing, and adhesive application are omitted with self-adhesive cement, thereby overcoming the technique and handling errors [20]. Moreover, the setting mechanism of RelyX Unicem self-adhesive cement permits setting with low shrinkage [21]. Contraction during the setting of the resin cement could produce stress within the cement layer, which might induce debonding or cracks in the ceramic over time [22]. However, self-adhesive cement also has some disadvantages, such as lower bond strengths compared to the cement that utilises the etch-and-rinse bonding system. [23]. Nevertheless, in the present study, it was attempted to simulate the worst-case scenario, in that the bond of resin cement to tooth structure was low. Furthermore, for ceramic surface treatment, a protocol was followed that provided a strong bond to ensure no failure from ceramic/cement interface. Therefore, veneers were etched with a 9.5% HF for 90 seconds that has been showed to increase the bond strength of lithium disilicate ceramic to resin cements [24–26].

Regardless of the polymerisation mode, the bonding of resin cement to another resin occurs through a mechanism of free-radical polymerisation and covalent bonding to the organic matrix. However, when a PLV is indicated on a tooth with an old composite filling, the bonding between the resin cement and composite may be compromised due to reduced unreacted monomer because of several degradation processes such as water sorption/solubility, pH action, and mechanical breakdown [27]. The greatest amount of free monomer is present on the surface of a composite filling during the first 24 h after polymerisation and decreases dramatically after one month [15]. In the present study, PLVs were bonded after storage in water for three weeks. Accordingly, minor or no free monomer could be expected on the surface of composite fillings. Unicem contains special methacrylate monomers with bonded phosphoric acid groups with at least two carbon double bonds. It is suggested that the acidic functional monomer in Unicem may ease penetration into the polymeric structure of a composite resin and mediate the acid-base setting reaction with the organic fillers available in the polymer matrix [22]. Simultaneously, radical polymerisation of methacrylate monomers through reactive carbon double bonds promotes copolymerisation with another polymer material [21].

Various surface treatments have been incorporated to improve the bond of new composite resin with an old one including sillicoating, chemical and mechanical roughening, and application of adhesive layers [28, 29]. However, the findings were not consistent. Furthermore, it was shown that the result is highly material-dependent [30]. Therefore, in the present study, the surface of composite fillings was ground using diamond burs, and no further treatment was applied to avoid extra confounding parameters. Surface treatments (e.g., airborne-particle abrasion, acid etching, silanization, and application of a low-viscosity adhesive) might improve the bonding of resin cement to old composites; therewith more favourable outcomes may be expected that could be investigated in future studies.

In the present study, we designed the intraenamel margins to simplify the research parameters. In the clinic, dentinal exposure is inevitable in most PLV preparations, particularly in the cervical area where the enamel is thin (<0.5 mm) [1, 12]. Thus, the effect of the dentinal margin in class V composite fillings should be tested in future studies.

In our study, laminate fracture was rare, and tooth fracture and mixed failure were the two most occurring failure modes in the NC and SC groups (Table 2). It could be argued that in teeth with larger-composite filling (LC), more enamel has been removed, and a decrease in the elastic modulus of the tooth could result in more distortion under loading and an increased rate of debonding. Early debonding may save the LC group from fracture and increase the frequency of adhesive and mixed failure [4, 31]. On the other hand, in the NC and SC groups, adhesive failure was rare. Higher fracture resistance in these groups may imply that the bond strength was high enough to withstand cyclic loading and eventually failure would occur cohesively within the cement layer and/or the tooth itself [4, 13, 14].

This was an in vitro study, and the findings cannot be directly extrapolated to the clinical situation. Nonetheless, several conditions were applied to fit the clinical situation. Thermal cycling and load cycles were applied to simulate certain conditions of real-life restorations. Natural teeth were selected because they have unique properties, such as elasticity, enamel, and dentin bonding properties, as well as a complex geometry of preparation, which may influence the findings of the study. However, we faced obstacles in terms of tooth availability and the standardisation of the tooth size and age. Nevertheless, the results should be regarded with caution as a perfect simulation was not possible. Finally, only one cement type was used in the present study. Further studies involving diverse types of resin cement and composite surface treatments should be performed.

## 5. Conclusion

Within the limitation of the present study, significantly stronger bonds were achieved in the control and small-composite groups compared to the large-composite group. This result was supported by the prevalence of cohesive failures in the control and small-composite groups. Adhesive failure was predominant in the large-composite group, confirming the weaker bond strength in this group.

## Figures and Tables

**Table 1:** The descriptive data for failure load values of test groups.

Test group	Number	Mean (*N*)	Standard deviation (*N*)	Min. (*N*)	Max. (*N*)	95% confidence interval	
						Lower bound	Upper bound
NC	11	364.28^a^	114.88	219.30	616.50	−296.46	432.10
SC	11	324.57^a^	89.25	220.10	471.4	−271.88	377.53
LC	10	207.26^b^	90.62	110.10	369.00	−151.05	263.47

Items in the rows with dissimilar letters are significantly different at 95% level of confidence; ^a^values are nonsignificantly different at *P* = 0.81; ^b^value is significantly different at *P* = 0.004.

**Table 2:** Failure modes of broken specimens.

Group	Cohesive fracture in tooth	Cohesive fracture in laminate	Adhesive	Mixed cohesive and adhesive
Control	5	1	0	5
SC	4	0	3	4
LC	1	0	5	4
Total	10	1	8	13
